# Dyad pedagogy in practical anatomy: A description of the implementation and student perceptions of an adaptive approach to cadaveric teaching

**DOI:** 10.1002/ase.2184

**Published:** 2022-05-27

**Authors:** Dearbhla P. Cullinane, Denis S. Barry

**Affiliations:** ^1^ Discipline of Anatomy Trinity College Dublin The University of Dublin Ireland

**Keywords:** anatomy pedagogy, blended learning in anatomy, dyad pedagogy, gross anatomy, student perception, undergraduate medical education

## Abstract

Prior to the challenges imposed by the Covid‐19 pandemic, anatomy practical sessions at Trinity College Dublin involved eight to 10 students per donor station, rotating between digital learning, anatomical models/osteology, and dissection activities for three hours weekly. To maintain cadaveric participation in the anatomy laboratory while adhering to distancing guidelines, a transition to dyad pedagogy was implemented. This mode of delivery allowed two students per donor station to spend one hour per week in the anatomy laboratory with all digital learning elements transferred to the virtual learning platform Blackboard as pre‐ and post‐practical session learning activities. Dyad pedagogy has been explored in clinical settings and simulation procedural‐based training but is yet to be fully verified in anatomy education. To determine the effectiveness of hybrid practical sessions and reduced donor to student ratios, the opinions of first year medical students were examined using an online questionnaire with a 51% response rate. Although students recognized the merits of more time in the anatomy laboratory, including opportunities for self‐directed study and exposure to anatomical variation, they felt that having two students per station enabled sufficient hands‐on time with the donor body and fostered learning opportunities that would not be possible with larger groups. Strong preferences for quality time with the donor body supported by online resources suggests this modality should be a key consideration in course design for anatomy curricula and emphasizes the importance of gauging students' preferences to optimize satisfaction and learning output when pivoting to blended learning strategies in anatomy education.

## INTRODUCTION

Cadaveric‐based teaching remains a key pedagogue in health science education due to its promotion of professionalism, ethical consciousness, and enhancement of communication skills (Flack & Nicholson, 2018). Notwithstanding, increasing student numbers, congested medical curricula, trends showing increases in transactional distances, and the strains of the recent Covid‐19 pandemic have resulted in a documented reduction in cadaveric contact time (Drake et al., 2009; Carmichael, 2012; Singh et al., 2015; Stone & Barry, 2019; Rockarts et al., 2020).

The Covid‐19 pandemic has accelerated a preexisting enthusiasm for anatomy educators to evaluate and challenge traditional approaches to pedagogy as measured by the increasing number of published articles related to anatomy education (Smith & Pawlina, 2021). Changes to anatomy curricula during the pandemic were emergency driven and many were reactive rather than proactive. Emergency responses saw anatomists adapt their conventional teaching approaches by delivering lecture content online and by adopting new synchronous and asynchronous online strategies to make‐up for lost contact time (Longhurst et al., 2020). Pather et al. (2020) indicated that such changes resulted in a loss of integrated “hands‐on” experiences that impacted academic workload, student roles, as well as anatomists' personal educational philosophies. Nonetheless, some evaluations of these pandemic‐driven pedagogical changes have been positive such as more time for self‐directed study and an increase in available blended learning resources (Srinivasan, 2020; Yoo et al., 2021).

To accommodate for social distancing guidelines and to ensure cadaveric participation in the anatomy laboratory was maintained, a transition to dyad pedagogy was implemented at Trinity College Dublin. Dyad pedagogy is a goal‐directed teaching method that arranges students in pairs and can be seen as both interactive and reciprocal in nature helping to accommodate the strengths and weaknesses of each member (Sherman & Márquez, 2006). Dyad pedagogy has been used in the area of simulation‐based procedural skills training for medical students and surgical residents, and as measured by procedural performance, dyad practice has been shown to be as effective as individual practice (Shanks et al., 2013; Räder et al., 2014; Tolsgaard et al., 2015; Kowalewski et al., 2019). Working in pairs in this setting has been shown to permit more efficient use of simulators, is more cost‐effective than individual practice, and in the case of laparoscopic cholecystectomy, reduces surgical operating time (Kowalewski et al., 2019). Notably, dyad training for procedural skills has also been shown to significantly reduce stress and anxiety among students while learning (Abbott et al., 2021).

The dyad approach can be seen as one that promotes collaborative learning and certainly, collaborative practices in health science education are becoming increasingly common, varied and generally well accepted (Pluta et al., 2013). In clinical settings, students are frequently encouraged to adopt and develop collaborative skills by participating in peer cooperation such as dividing learning tasks among peers, peer monitoring such as observing, and peer tutoring such as researching relevant topics and teaching them to each other (Sevenhuysen et al., 2016). As a collaborative approach, dyad pedagogy has been explored widely in nursing programs as a method of improving the quality and efficiency of clinical instruction and for creating supportive learning environments (Ruth‐Sahd, 2011; Austria et al., 2013; Ott & Succheralli, 2015). In a study by Ott and Succheralli (2015), where student nursing dyads were expected to provide complete care to their assigned patient by functioning as a team, students reported that the dyad system had had a positive impact on their experiences of teamwork and clinical confidence. Other studies showed that working in dyads reduced student anxiety, increased confidence and task efficiency, improved patient outcomes, and helped to instill very early in the education process the importance of teamwork (Ruth‐Sahd, 2011; Austria et al., 2013). More recent studies have expanded upon this design by formulating interprofessional learning dyads, that is medical student–nursing student pairs. Preliminary research in the area has shown that working in interprofessional dyads helps medical students gain an awareness of their profession's strengths and weaknesses and can lead them to a more holistic understandings of treatment (Hansen et al., 2020).

But what of the cognitive aspects of dyad pedagogy? Cognitive load theory may help to explain the advantage of using dyads as a process that unites memory and collaborative information processing (Kirschner et al., 2009; Räder et al., 2014). Complex tasks, such as memorizing a great amount of anatomical detail during a practical session, risks overloading the learner's working memory. By collaborating with a partner, however, this load is shared and therefore lessened for each individual. The exercise of collaboration itself, however, may *increase* cognitive load. The product of the two is a cognitive load equilibrium, but this balance may be greatly swayed by other elements such as gender, personality congruence, relationships between dyads, and previous experience (Xue et al., 2018; Wang et al., 2022). One way of objectively measuring collaborative behavior has been via interpersonal brain synchronization studies. Sun et al. (2021) for example studied the effect of member experience on dyad cooperation using functional near‐infrared spectroscopy‐based hyperscanning. Student–student dyads and teacher–student dyads were examined. The results revealed that members with differing experiences (teacher‐student dyads) performed better on a joint‐drawing task than those with similar experiences (student–student dyads), and interpersonal brain synchronization of the left frontopolar region was found in teacher–student dyads only. Another study by Xue et al. (2018) which compared interpersonal brain synchronization between highly creative (high) and less creative (low) individuals when solving realistic presented problems, found that dyads consisting of two low‐creativity members could perform just as well as dyads of two high‐creativity members. Moreover, stronger interpersonal brain synchronization between group members was evoked in low–low dyads, suggesting that better cooperation results in enhanced performance. These studies provide valuable insights for real‐world dynamics where people must collaborate effectively.

For anatomy, the concept of dyad pedagogy has been examined at Downstate Health Sciences University, New York (Sherman & Márquez, 2006; Márquez & Sherman, 2009; Noronha et al., 2010; Blumenberg et al., 2011; Blumenberg & Márquez, 2012). One area in particular has looked at the integration of dyad pedagogy and technology to bolster anatomy learning by having student dyads create video projects in the anatomy laboratory. With access to a myriad of learning modalities including textbooks, lecture slides, and the internet, students define muscles and innervations of a particular region of the body and explain issues associated with injury to these structures. The presentation is then video‐recorded using high‐definition recording devices and thereafter posted to the Intranet (a private online network) for their classmates to use during review and study (Noronaha et al., 2010). The result is an online atlas of anatomy videos with clinical insights that augment the learning of gross anatomy (Blumenbery & Márquez, 2012). As measured by online activity logs, their value is evidenced by the frequency in which these videos are viewed, specifically when approaching examination periods. About 25% of medical students were found to view the videos five times or more and usage increased substantially in the days before an exam, implying active utilization of the videos as a study tool. In their approaches, dyad pedagogy has been considered a powerful method for acquiring and integrating anatomical knowledge that students can take beyond the classroom and into the workplace. These include problem‐solving skills, research, oral and written presentation, decision‐making, judgment, working collaboratively, and an ability to self‐learn (Sherman, 2008).

With evidence‐based pedagogy now at the forefront of anatomy education (Evans et al., 2020; Smith & Pawlina, 2021), evaluating student preference should be considered a constructive tool for designing anatomy curricula (Davis et al., 2014; Phillips et al., 2018). Dyad pedagogical approaches may be particularly applicable for anatomy practical sessions that relate to the axial skeleton, due to the unilateral and/or central nature of these regions, that is, one trachea, one heart, one liver, one bladder. By comparison, larger groups may be more appropriate in practical sessions that explore musculoskeletal regions. Dyad pedagogy may however limit experiences of variant anatomy and restrict interactions among peers, both of which are important practices for the future doctor (Sprunger, 2008; Cullinane & Barry, 2022).

Although dyad pedagogy has been explored in procedural skills, clinical settings, and as a method of optimizing resources in a high‐technology enabled anatomy laboratory, it remains unknown whether this pedagogical method is a suitable substitute to small group learning and whether such an approach is beneficial when supplemented with online blended learning resources. Moreover, the benefits of small group anatomy practical sessions from the student's perspective are unclear. This study describes the process of pivoting to a blended thorax, abdomen, and pelvis anatomy practical session curriculum in response to social distancing guidelines while maintaining adequate cadaveric contact time in the anatomy laboratory via the dyad pedagogical approach.

## MATERIALS AND METHODS

The study sought to quantify medical student opinion of pair learning for cadaveric thorax, abdomen, and pelvis anatomy practical sessions at Trinity College Dublin. The study design relates to level 1 of the Kirkpatrick model for evaluating training programs and therefore assesses the degree to which participants find anatomy practical sessions engaging and relevant and aims to measure students' initial reactions to dyad pedagogy (Kirkpatrick & Kirkpatrick, 2006). First year medical students voluntarily responded to an anonymized, self‐administered online questionnaire via Qualtrics Survey Tool (Qualtrics, Provo, UT). Participants were informed that no personally identifiable information would be associated with their responses and that they may withdraw at any time by closing the web browser. The School of Medicine Research Ethics Committee Trinity College Dublin granted approval for the use of survey data in this study. Approval number 20210206.

### Course structure

At Trinity College Dublin, the anatomy curriculum for medical students is delivered over four modules and takes a regional‐based approach. Two modules are taken during the first year: musculoskeletal anatomy (September–December), and thorax, abdomen, and pelvis anatomy (January–April). In the second year, a further two modules are taken; anatomy of the head and neck in the fall, followed by neuroanatomy in the spring. Over the two years, approximately 200 hours of anatomy content is delivered with 25% delivered as didactic lectures and 75% delivered as practical sessions in the anatomy laboratory. The anatomy practical sessions are primarily dissection based in which small groups of students dissect a cadaver under the supervision of an anatomy demonstrator. Students are alphabetically assigned to groups by the senior executive officer to the discipline and students remain in these groups for one year. Other teaching resources such as models, osteological specimen, radiological images, and digital learning platforms are also used. In general, 180–200 students are enrolled in the course each year and 182 students entered the medical program in 2020.

### Practical teaching and assessment of thorax, abdomen, and pelvis anatomy

#### Prior to the Covid‐19 pandemic

**FIGURE 1:**
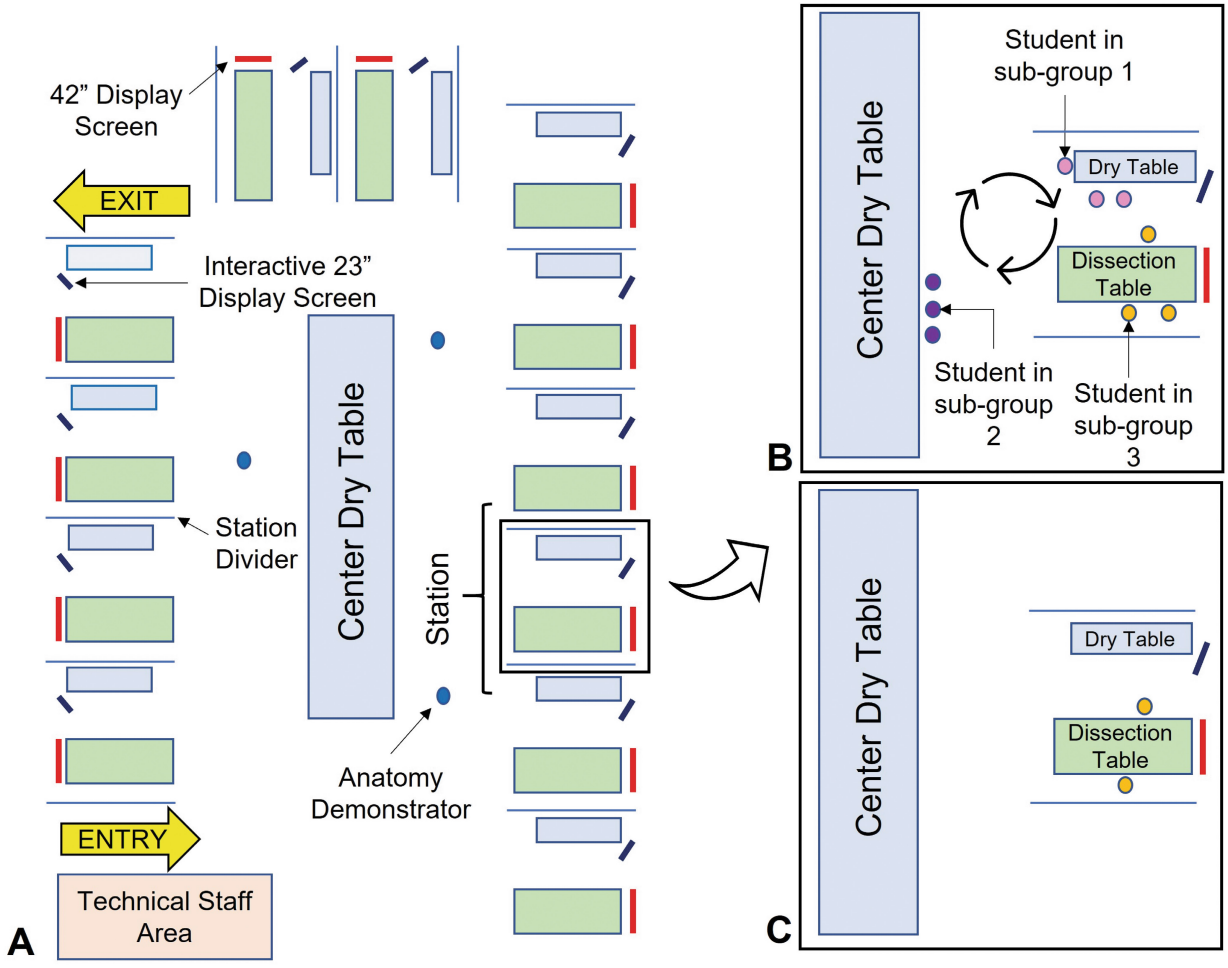
(A) The layout of the anatomy laboratory during anatomy practical sessions at Trinity College Dublin. The anatomy laboratory is comprised of 12 stations with each station occupying a cadaveric dissection table, a dry table for variable learning using models, osteological specimen, and anatomy atlases, one 42‐inch display screen, and one 23‐inch interactive display screen. Each station is separated using station dividers. The anatomy demonstrators float between stations and guide students who are engaged in self‐directed study. (B) Example of station layout prior to the Covid‐19 pandemic. Each station has eight to ten students. Students are divided into subgroups and rotate between cadaveric dissection, digital learning and dry‐table learning activities for three hours. The ratio of faculty to students is one anatomy demonstrator to approximately 36 students. (C) Example of layout during the Covid‐19 pandemic. A list of anatomical structures to be identified during the one hour session are displayed on the 42″ display screen at each table. Anatomical models are stationed at the “Center Dry Table.” Students may bring desired models to their respective dry tables and place back on the center dry table at the end of the session. The ratio of students per anatomy demonstrator is eight students per one anatomy demonstrator

The anatomy laboratory at Trinity College Dublin is comprised of 12 stations with each station occupying a cadaveric dissection table, a dry table for variable learning using models, osteological specimen, and anatomy atlases, one 42‐inch display screen, and one 23‐inch interactive display screen. Each station is separated using station dividers and the layout is depicted in Figure 1. Prior to the Covid‐19 pandemic, the thorax, abdomen, and pelvis anatomy curriculum at Trinity College Dublin was delivered as a 12‐week course comprised of 11 three‐hour practical sessions and 20 one‐hour didactic lectures. Practical sessions were comprised of eight to ten students per station and involved small subgroup rotations between cadaveric dissection, digital learning, and dry‐table learning activities (Figure 1B). The use of station‐based rotations in the anatomy laboratory has previously been reported in the literature and is noted for maximizing student engagement and for providing students with multiple means of representation (Drake, 2007; Goldina & Barattini, 2018; Balta et al., 2021). Cadaveric dissection involved a subgroup of approximately three students participating in dissection of a donor body for one hour using a designated dissection manual that was uploaded to the virtual learning platform and displayed on the 42‐inch monitor during the practical session. Assistance and supervision was provided by an anatomy demonstrator. Digital learning involved another subgroup of students engaging in an interactive PowerPoint presentation (Microsoft Corp., Redmond, WA) on a 23‐inch display screen. The PowerPoint presentation involved cadaveric and radiological images and asked students to work as a team to identify anatomical structures and relate their knowledge to clinically relevant scenarios (O'Keeffe et al., 2019). The final dry‐table rotation at the center of the anatomy laboratory involved the use of anatomical models, osteological specimen and atlases to review anatomical content. During this rotation, students have the liberty to use these resources as they so wish. Each of the rotations was weighted at one hour and faculty cover of the anatomy laboratory was one anatomy demonstrator to four stations (approximately 36 students). Furthermore, students had the opportunity to engage in additional self‐directed study in the anatomy laboratory outside designated class time.

Student performance of practical anatomy was measured using traditional ‘anatomy spot examinations’ housed in the anatomy laboratory followed by an end‐of‐module multiple choice questionnaire. Students completed three in‐house ‘anatomy spot examinations’. The first two were continuous assessments and completed during weeks four and nine of the module with each assessment accounting for 10% of the final grade. Each of these continuous assessments were comprised of five questions with four parts; parts 1 and 2 asked students to identify anatomical structures tagged on cadaveric material; part 3 asked students to provide information regarding arterial supply, venous drainage, innervation, function, embryological origin, and/or anatomical relations; and part 4 assessed students' ability to apply clinical knowledge. Each part was weighted with one mark summing to a total potential mark of 20. Students were allocated four minutes per question. The third and final anatomy spot examination was held during week 12 and was comprised of ten questions with five parts; parts 1 to 4 followed the same format as the continuous assessments and part 5 ranged from basic identification of anatomical structures to clinically applied anatomy. Each part was once again weighted with one mark summing to a total potential mark of 50 and accounting for 40% of the total grade. Five minutes were allocated per question. Lastly, students completed an end‐of‐module 50‐item multiple choice questionnaire that accounted for 40% of the final grade and was 90 minutes in duration.

#### During the Covid‐19 pandemic

The thorax, abdomen, and pelvis anatomy module 2021 was adjusted in response to social distancing and was divided into three units, thorax (3 weeks), abdomen (4 weeks), and pelvis (1 week), followed by 1 week of self‐directed revision. Students were assigned to dyads and one hour per week, for eight consecutive weeks, was spent in the anatomy laboratory (two students per station). For anatomy laboratory layout with social distancing guidelines see Figure 1C. Cadavers were “semi‐prosected” by anatomy faculty prior to attendance by students, that is, cadavers were dissected so that all major anatomical landmarks were visible and students were given the opportunity to participate in more detailed dissection to expose smaller structures. After the 1‐h practical, a new set of student pairs entered the anatomy laboratory and continued the dissection that had been completed by students prior. Students were able to revisit this regional dissection for the first 5–10 minutes of the following week. Faculty cover of the anatomy laboratory was one anatomy demonstrator to four stations (eight students) with the total number of students in the anatomy laboratory per practical session summating to 24 students. The lecture delivery, which covers basic anatomy, clinical relations, and embryological development was presented in the same pre‐Covid‐19 structure albeit online using a combination of prerecorded and live lectures using Panopto video hosting platform (Panopto Inc., Seattle, WA) and Collaborate Ultra (Blackboard Inc., New York, NY). To compensate for the reduction of time spent in the anatomy laboratory, pre‐ and post‐practical session learning activities were uploaded to the virtual learning platform Blackboard. The pre‐practical session activity included links to “*Acland's Video Atlas of Human Anatomy*” (Acland, 2013) and a detailed pre‐practical guide that listed the aims and objectives of the practical session as well as a comprehensive list of anatomical structures to be identified during the 1‐h practical session. The post‐practical session activity substituted the digital learning element that was provided during practical sessions pre‐Covid‐19 and included a self‐test PowerPoint presentation (Microsoft Corp., Redmond, WA) which asked students to label diagrams, review radiological images, and relate their anatomical knowledge to clinically relevant scenarios.

Student performance of practical and theoretical anatomy followed the same pre‐Covid‐19 format, however, these assessments were transferred to the virtual learning environment. The traditional in‐house “spot anatomy examinations” were substituted with cadaveric images and students were assessed on their ability to identify, relate, and clinically evaluate anatomical content. The end‐of‐module multiple choice questionnaire was also transferred online to the virtual learning environment. The same time allowances were allocated and all online assessments were remotely proctored using Proctorio, a remote proctoring service (Proctorio Inc., Scottsdale, AZ).

### Search strategy to identify available instrument

A systematic literature search of PubMed (United States National Library of Medicine, National Institutes of Health, Bethesda, MD) (1988–2022) and Embase^®^ (Elsevier, Inc., New York, NY) (1970–2022) attempted to identify articles relevant to dyad pedagogy and student satisfaction in anatomy. Key words used in the PubMed search were re‐executed in Embase^®^. All articles that matched our search terminology failed to identify a survey instrument that addressed the specific evaluation needs. A valid and reliable instrument to measure student perceptions of dyad pedagogy in practical anatomy was therefore developed.

### The questionnaire

The first part of this questionnaire gathered demographic data including gender, age, previous anatomy and dissection experience, future career interest, and the number of anatomy practical sessions attended by the participant (six items). Using a five‐point Likert scale, the second part asked participants to what extent they agreed with a series of statements regarding students' preparedness for practical sessions, feelings of connectedness to faculty and peers, usefulness of accompanying online learning resources, and the extent to which they agreed with the mode of assessment (1 = strongly disagree, 2 = disagree, 3 = neither agree nor disagree, 4 = agree, 5 = strongly agree; 10 items). The third part asked students whether they had seen or missed anatomical structures during the practical sessions as a measure of the amount of detailed dissection achieved (two items; Selçuk et al., 2019). Lastly, the final section asked students whether they enjoyed the pair‐based system (one item), and a space was provided for participants to express supplementary thoughts and opinions. The questionnaire was modeled on items previously published in anatomy student perception studies (Vasan et al., 2009; Jeyakumar et al., 2020). The Cronbach's alpha coefficients for Vasan et al. (2009) and Jeyakumar et al. (2020) were 0.908 and 0.810, respectively, and the original “Pair‐Learning in Practical Anatomy Survey” is available in the Supporting Information File.

### Data analysis

Reliability of the questionnaire was assessed using Cronbach's alpha coefficient; values greater than 0.7 were considered acceptable (Peterson, 1994; Santos, 1999). Cronbach's alpha is a statistical measure of internal consistency that ranges from 0 to 1. As the statistic approaches 1, a greater degree of internal consistency between items in the Likert scale is indicated and therefore signifies reliability of the instrument. The Kendall's tau‐b coefficient was used to assess the validity of the questionnaire. Kendall's tau‐b is a nonparametric rank correlation coefficient that measures the strength of the association between sets of paired data. Significant tau‐b coefficients indicate construct validity of the questionnaire (Sokal & Rohlf, 1995). Descriptive summary statistics (frequencies and percentages) were calculated for basic demographic data.

A principal components analysis with varimax rotation was conducted to identify sub‐measures within the pair learning in practical anatomy questionnaire. Varimax rotations maximize the sum of the variances within a model and helps to clarify relationships among factors. It is the most frequently reported rotational method used in published studies (Thompson, 2004). The Kaiser–Meyer–Olkin (KMO) measure of sampling adequacy and the Bartlett's test of sphericity were used to determine whether the analysis should proceed with exploratory factor analysis. The KMO index ranges from 0 to 1, with KMO >0.50 considered necessary for factor analysis. Similarly, Bartlett's test of sphericity should be statistically significant (*P* < 0.05; Williams et al., 2010). Factors that yielded eigenvalues greater than 1 were retained as factors within the model (Kaiser, 1960; Taherdoost, 2016). Items with cross‐loadings, that is loadings of 0.3 or above on two factors, were eliminated. Reliability of the factors was assessed using Cronbach's alpha coefficient (Peterson, 1994; Santos, 1999).

Likert‐scale responses often depart from the normal distribution, the Mann–Whitney *U* test was therefore utilized to compare responses between students with previous cadaveric anatomy experience versus those without, and between students interested in surgical/radiological careers or other specialties. As the Mann–Whitney *U* test is an ordinal test, medians are recommended as the reported measure of central tendency (Field, 2009), however, means and standard deviations are also reported here. To evaluate the effect size of any significant differences observed in the Mann–Whitney *U* test the correlation coefficient (*r*) was calculated with *r* > 0.10 representing a small effect size, *r* > 0.3, medium; and *r* > 0.5, large (Rosenthal, 1991). Differences in responses between age groups and between genders were examined using the Kruskal–Wallis *H* test. The Kruskal–Wallis *H* test, also known as the “One‐Way ANOVA on Ranks” is a nonparametric test used to compare two or more independent samples of equal or different size by comparing median values. It is particularly suitable for ordinal data and where there is a considerable difference in the number of subjects for each comparative group (MacFarland & Yates, 2016). Dunn post‐hoc tests with Bonferroni adjustments were performed for statistically significant Kruskal–Wallis values. The Dunn post‐hoc test is a nonparametric pairwise multiple comparisons procedure based on ranked data and is recommended for groups with unequal sample sizes (Elliott & Hynan, 2011). Significant values (*P* < 0.05) indicate differences between groups. Bonferroni adjustments correct for multiple comparisons and are recommended to avoid the occurrence of type I errors (Armstrong, 2014). Effect sizes were calculated using eta‐squared; eta‐squared < 0.01 small, eta‐squared < 0.06 medium, and eta‐squared < 0.14 large. Differences of statistical significance were set as *P* < 0.05. Statistical Package for Social Sciences (SPSS), version 26 (IMB Corp., Armonk, NY) was used for analysis of quantitative data.

The open‐ended qualitative responses were collated, and an inductive content analysis was performed. Inductive content analysis is used in cases where there are no previous studies dealing with the phenomenon (Elo & Kyngäs, 2008). The two authors independently open coded the responses. This involved the process of reading text and writing down headings in the margins to describe all aspects of the content (Bernard, 1996). The independent headings formulated by the authors were thereafter collated from the margins by the lead investigator to form categories and each category was named using a content‐characteristic word. These categories were further refined by constructing subcategories. The identified categories were reviewed by the second author and relevant categories were retained.

## RESULTS

### Questionnaire validity and reliability

The Cronbach's alpha value of 0.75 indicated an acceptable correlation coefficient for the cumulative Likert‐scale items. The alpha value, which is over the 0.70 threshold indicates that the instrument concerning student satisfaction of pair learning in practical anatomy is reliable. Significance testing of the Kendall's tau‐b statistic showed significant positive associations *P* < 0.01) between all items. Coefficients ranged from 0.216 to 0.561. The results demonstrate validity of the survey instrument (Sokal & Rohlf, 1995). Scores ranged from 28 to 49 with higher scores indicating greater satisfaction with pair learning in practical anatomy sessions.

### Survey response and cohort characteristics

Ninety‐three first year medical students voluntarily participated in the study (51% response rate). Females accounted for 73.1% (*n* = 68) of the study population with males accounting for 25.8% (*n* = 24). One participant identified as other (1.1%, *n* = 1). Eighty‐one percent of participants fell within the 17–20‐year‐old category (*n* = 76), 14% in the 21–24‐year‐old category (*n* = 13), and 4.3% were aged 25 to 28 (*n* = 4). Approximately 9% of participants had had previous experience with cadaveric anatomy and all participants (100%) attended more than 50% of the practical sessions in the anatomy laboratory indicating regular attendance. Approximately half of all participants surveyed indicated that they would be interested in a surgical or radiological career (52.7%). Twenty‐six participants (27.95%) contributed to the open‐ended responses.

### Exploratory factor analysis

A principal component factor analysis with varimax rotation was conducted on the 10 items of the pair learning in practical anatomy questionnaire. The Kaiser–Meyer–Olkin measure of sampling adequacy was 0.683, which surpassed the 0.5 threshold and the Bartlett's test of sphericity was statistically significant (*P* < 0.001). Exploratory factor analysis was therefore performed. Three factors with eigenvalues greater than 1.0 were identified and found to explain 32%, 15%, and 12% of the variance, respectively. An initial five items were eliminated due to cross‐loadings of 0.3 or above. The item “*During the pair‐based anatomy practicals, I had enough face‐to‐face time with my demonstrator*” had factor loadings between 0.4 and 0.6 on both Factor 1 and Factor 3; “*My anatomy lab partner and I worked well together*” had factor loadings between 0.5 and 0.7 on both Factor 1 and Factor 2; and “*Given the one‐hour pair‐based anatomy practical sessions, I still feel the practical examination provides a fair assessment*” had factor loadings between 0.4 and 0.7 on both Factor 1 and Factor 3. The principal components analysis with varimax rotation of the revised seven‐items was rerun. Two factors with eigenvalues greater than 1.0 were identified and were determined to explain 50.80% of the variance. An additional item was eliminated due to cross‐loadings; item “*I was well prepared for my practical each week*” had factor loadings between 0.4 and 0.5 on both Factor 1 and Factor 2.

For the final stage, a principal component factor analysis of the remaining six items, using varimax rotation was conducted. Two factors explained 54.42% of the variance. All items in this analysis had primary loadings over 0.5. Only one item had a cross‐loading above 0.3 (pair learning helped my understanding of anatomy), however, this item had a strong primary loading of 0.698 on the second factor. The factor loading matrix for this final solution is presented in Table 1. Follow‐up reliability analysis of the identified factors extracted poor Cronbach's alpha coefficients; 0.552 for Factor 1 (3 items) and 0.410 for Factor 2 (3 items). The six items comprising the two factors were determined to represent “*Connectedness and Preparedness*” and “*Understanding and Online Learning*”; however, no follow‐up comparisons analyses were performed on either of the two constructs due to insupportable reliability.

**TABLE 1:** Factor loadings and communalities based on principal components analysis with varimax rotation for six items from the pair learning in practical anatomy questionnaire

Rotated component Matrix^a^	Factor		Communality^g^
	1^e^	2^f^	
My anatomy lab^b^ partner was well prepared for the practical each week	0.801		0.641
Given the paired nature of the anatomy practicals^c^, I still feel connected to my other classmates	0.677		0.458
During the pair‐based anatomy practicals^c^, I had enough hands‐on time with the donor body (cadaver) at my station	0.673		0.468
I learn better from the online lectures than in my pair‐based anatomy practicals^c^		−0.785	0.642
Pair‐based learning helped my understanding of anatomy	0.459	0.698^h^	0.698
The pre‐ and post‐lab^d^ learning activities on Blackboard were useful tools for private study and revision		0.596	0.358

*Note*: Extraction Method: Principal Component Analysis. Rotation Method: Varimax with Kaiser Normalization. Number of respondents (*n* = 93).

Factor loadings <0.3 are suppressed.

^a^ Rotation converged in three iterations.

^b^ Lab, laboratory.

^c^ Practicals, practical sessions in the anatomy laboratory.

^d^ Pre‐lab, pre‐practical session learning activity on virtual learning platform Blackboard; post‐lab, post‐practical session learning activity on Blackboard.

^e^ Factor represents “Connectedness and Preparedness,” Cronbach's alpha = 0.552.

^f^ Factor represents “Understanding and Online Learning,” Cronbach's alpha = 0.410.

^g^ The closer the communality is to 1, the better the variable is explained by the factors.

^h^ Primary loading for item, loaded on factor 2.

### Perceptions about connectedness and preparedness in pair‐learning given reduced time in the anatomy laboratory

**FIGURE 2:**
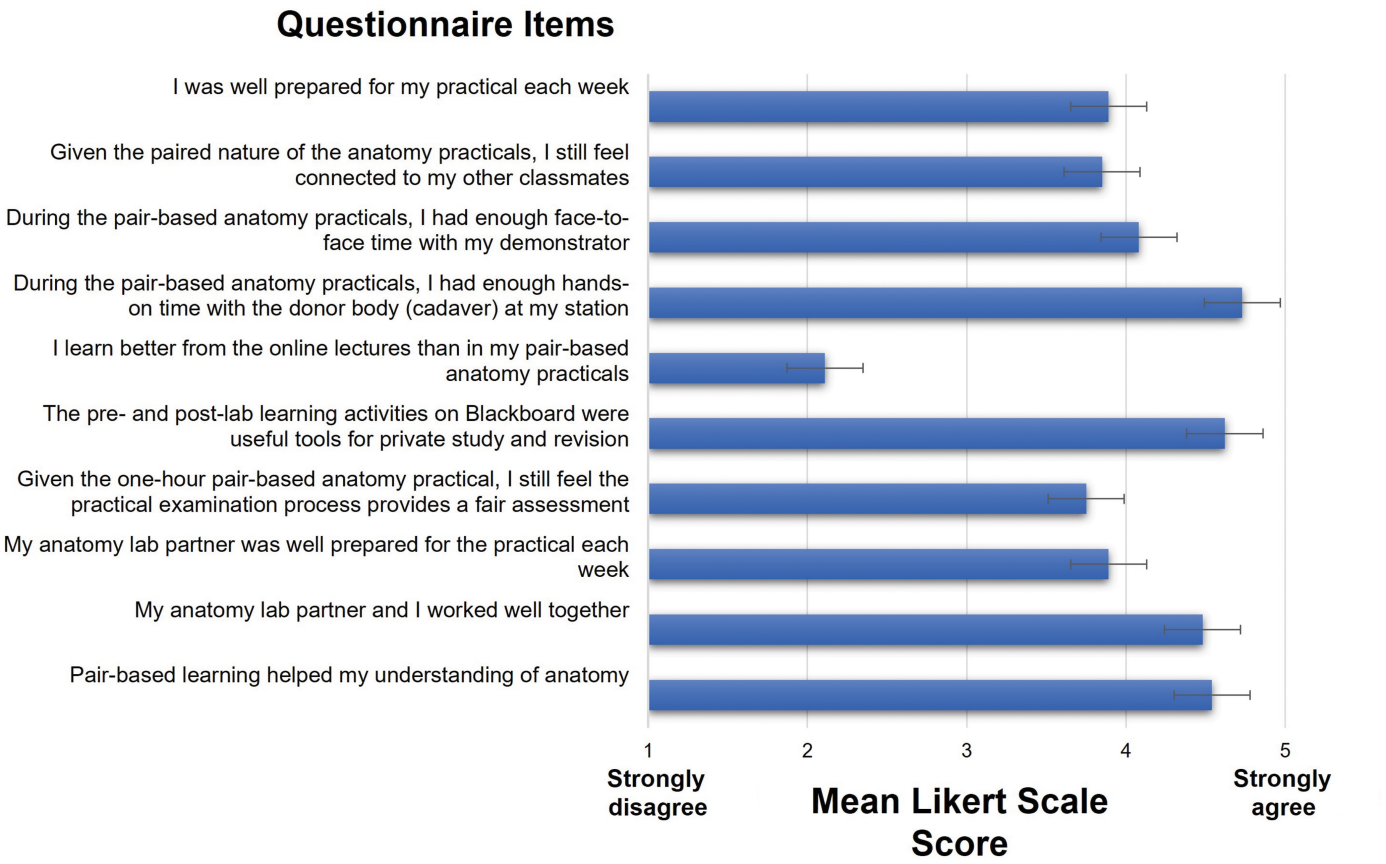
Mean Likert score responses to questionnaire items. Graphs showing the distribution of preferences (indicated by the bars) for a particular questionnaire item. Bars represent ± SEM, standard error of the mean. A Five‐point Likert scale was used, where 1 = strongly disagree, 2 = disagree, 3 = neutral, 4 = agree, and 5 = strongly agree

Despite a significant reduction in time allocated to the anatomy laboratory in comparison to previous years, students appeared to maintain sufficient relationships with their peers and demonstrators. Students agreed that pair learning helped their understanding of anatomy (4.54 ± 0.69) and that they and their partner worked well together (4.48 ± 0.34). Figure 2 illustrates the mean Likert‐scale scores for the questionnaire items. Importantly, students concurred that they had enough time with the donor body at their stations (4.73 ± 0.68) indicating that reduced time with donors does not impact students' ability to build an appropriate professional relationship with their donor. Likewise, reduced contact with the donor body does not impact students' ability to dissect and identify small anatomical structures when skin and fascia have been readily dissected. This is reaffirmed with the third part of the questionnaire which asked students whether they had seen or not seen the left anterior descending artery and the major duodenal papilla during practical sessions. More than half of the students (64.5%) reported that they saw the left anterior descending artery on the donor body at their station. Notwithstanding, only 44.1% indicated that they saw the major duodenal papilla.

With reduced time in the anatomy laboratory at the forefront of this pair‐learning strategy, students were asked to respond to a series of statements concerning preparedness for practical sessions. As indicated in Figure 2, students reported that both they (3.89 ± 0.70) and their partners (3.89 ± 0.95) were well‐prepared for the practical sessions each week supporting the notion that reduced time promotes proactive learning. There was also agreement among students that the online‐practical examination provided a fair assessment (3.75 ± 0.96).

### Perceptions about online learning as an alternative to rotational‐based practical sessions

There was consensus among students that the pre‐ and post‐practical session learning activities on Blackboard were useful tools for private study and revision (4.62 ± 0.64). That said, however, students reported that they learned better during practical sessions as compared to online lectures (item 5; 2.11 ± 0.96) which highlights the notion that not all learning material can be effectively substituted online. As shown in Table 2, students with previous cadaveric anatomy experience (1.38 ± 0.52) rated item 5 significantly lower than those with no previous experience (2.18 ± 0.97) suggesting that students with no prior experience are more welcome to the idea of substituting in‐person anatomy laboratory time with online resources.

**TABLE 2:** Comparison of individual items for previous cadaveric anatomy experience

Item	Group						Mann–Whitney *U* test			
	Whole sample^a^		Previous cadaveric anatomy experience							
			Yes (*n* = 8)		No (*n* = 85)		*U*‐value	*Z*	*P*‐value	*r*
	Mdn	Mean (±SD)	Mdn	Mean (±SD)	Mdn	Mean (±SD)				
I was well prepared for my practical each week	4	3.89 (±0.70)	4	4.00 (±0.00)	4	3.88 (±0.73)	324.0	−0.278	0.78	NC
Given the paired nature of the anatomy practicals, I still feel connected to my other classmates	4	3.85 (±1.05)	4	3.88 (±1.13)	4	3.85 (±1.05)	335.0	−0.073	0.94	NC
During the pair‐based anatomy practicals, I had enough face‐to‐face time with my demonstrator	4	4.08 (±0.99)	4	4 0.0 (±1.07)	4	4.08 (±0.99)	325.5	−0.230	0.82	NC
During the pair‐based anatomy practicals, I had enough hands‐on time with the donor body (cadaver) at my station	5	4.73 (±0.68)	5	4.88 (±0.35)	5	4.72 (±0.70)	313.0	−0.539	0.59	NC
I learn better from the online lectures than in my pair‐based anatomy practicals	2	2.11 (±0.96)	1	1.38 (±0.52)	2	2.18 (±0.97)	169.0	−1.260	**0.01**	0.457
The pre‐ and post‐lab learning activities on Blackboard were useful tools for private study and revision	5	4.62 (±0.64)	5	4.88 (±0.35)	5	4.60 (±0.57)	265.0	−1.260	0.21	NC
Given the one‐hour pair‐based anatomy practical, I still feel the practical examination process provides a fair assessment	4	3.75 (±0.63)	4	4.13 (±0.36)	4	3.72 (±0.96)	268.0	−1.058	0.29	NC
My anatomy lab partner was well prepared for the practical each week	4	3.89 (±0.95)	4	4.0 (±0.54)	4	3.88 (±0.98)	339.0	−0.015	0.99	NC
My anatomy lab partner and I worked well together	5	4.48 (±0.64)	5	4.63 (±0.52)	5	4.47 (±0.65)	305.0	−0.544	0.59	NC
Pair‐based learning helped my understanding of anatomy	5	4.54 (±0.69)	5	4.88 (±0.35)	5	4.51 (±0.70)	246.5	−1.507	0.13	NC

Bold value represents statistically significant difference between groups.

^a^ Whole Sample, Entire group of students who participated in this study; Mdn, Median; ±SD, ±standard deviation; *r*, Pearson's correlation coefficient (effect size) *r* > 0.10 small effect size, *r* > 0.3, medium; and *r* > 0.5, large; NC, Not calculated.

### Comparisons analysis

The perceptions of students toward pair learning in practical anatomy were compared across career interest and previous cadaveric anatomy experience. Pair‐learning satisfaction scores for students with previous cadaveric anatomy experience (median = 44.50; mean = 43.86 ± 1.96) did not differ significantly from students with no previous experience (median = 42; mean = 41.53 ± 4.67), *U* = 244.50, *P* = 0.19. Likewise, no differences were observed between students interested in surgical/radiological careers (median = 43; mean = 41.71 ± 4.62) as compared to students interested in other medical disciplines (median = 42; mean = 41.75 ± 4.52), *U* = 1062, *P* = 0.90. The breakdown of responses for students with previous cadaveric anatomy experience versus those with no previous experience is summarized in Table 2. Although statistically insignificant (*P* > 0.05), all students with previous cadaveric anatomy experience rated all items higher than those with no previous experience. This excluded item 5, “I learn better from the online lectures than in my pair‐based anatomy practical sessions,” with those with previous experience rating the item lower (median = 1, mean = 1.38 ± 0.52) than those with no previous experience (median = 2, mean = 2.18 ± 0.97). This difference was statistically significant and represented a medium effect size *U* = 169, *P* = 0.01, *r* = 0.457. The breakdown of responses for individual items for students interested in surgical/radiological careers versus those interested in other disciplines is summarized in Table 3. No significant differences were observed.

**TABLE 3:** Comparison of individual items for future career interest

Item	Group						Mann–Whitney *U* test			
	Whole sample^a^		Future career interest							
			Radiological/surgical (*n* = 49)		Other discipline (*n* = 44)		*U*‐value	*Z*	*P*‐value	*r*
	Mdn	Mean (±SD)	Mdn	Mean (±SD)	Mdn	Mean (±SD)				
I was well prepared for my practical each week	4	3.89 (±0.70)	4	3.88 (±0.75)	4	3.91 (±0.64)	1074.5	−0.034	0.97	NC
Given the paired nature of the anatomy practicals, I still feel connected to my other classmates	4	3.85 (±1.05)	4	3.84 (±1.03)	4	3.86 (±1.09)	1043.5	−0.283	0.78	NC
During the pair‐based anatomy practicals, I had enough face‐to‐face time with my demonstrator	4	4.08 (±0.99)	4	4.00 (±0.98)	4	4.16 (±1.01)	963.5	−0.954	0.34	NC
During the pair‐based anatomy practicals, I had enough hands‐on time with the donor body (cadaver) at my station	5	4.73 (±0.68)	5	4.67 (±0.77)	5	4.80 (±0.55)	1005.0	−0.813	0.42	NC
I learn better from the online lectures than in my pair‐based anatomy practicals	2	2.11 (±0.96)	2	2.16 (±1.14)	2	2.05 (±0.71)	1056.0	−0.183	0.89	NC
The pre‐ and post‐lab learning activities on Blackboard were useful tools for private study and revision	5	4.62 (±0.64)	5	4.59 (±0.73)	5	4.66 (±0.53)	1062.5	−0.146	0.88	NC
Given the one‐hour pair‐based anatomy practical, I still feel the practical examination process provides a fair assessment	4	3.75 (±0.63)	4	3.80 (±1.02)	4	3.70 (±0.90)	982.5	−0.788	0.43	NC
My anatomy lab partner was well prepared for the practical each week	4	3.89 (±0.95)	4	3.94 (±0.92)	4	3.84 (±0.99)	1009.5	−0.569	0.57	NC
My anatomy lab partner and I worked well together	5	4.48 (±0.64)	5	4.53 (±0.58)	5	4.43 (±0.70)	1017.5	−0.528	0.60	NC
Pair‐based learning helped my understanding of anatomy	5	4.54 (±0.69)	5	4.63 (±0.70)	5	4.43 (±0.66)	869.5	−1.906	0.06	NC

^a^ Whole Sample, Entire group of students who participated in this study; Mdn, Median; ±SD, ±standard deviation; *r* ^2^, Pearson's correlation coefficient (effect size); NC, Not calculated.

A Kruskal–Wallis *H* test showed that there was no statistically significant difference in pair‐learning satisfaction scores between age groups *χ* ^2^(2) = 0.577, *P* = 0.75, with a mean rank satisfaction score of 46.04 for 17 to 20 year olds (*n* = 76), 52.08 for 21 to 24 year olds (*n* = 13), and 48.75 for 25 to 28 year olds (*n* = 4). However, similar to the results found for previous cadaveric anatomy experience, using the Kruskal–Wallis *H* test an analysis of responses for individual items across age groups indicated a significant difference for item 5 “I learn better from the online lectures than in my pair‐based anatomy practical sessions” *χ* ^2^(2) = 8.502, *P* = 0.01, with a mean rank score of 50.57 for 17 to 20 year olds, 31.35 for 21 to 24 year olds, and 30.00 for 25 to 28 year olds. Pairwise comparisons using the post‐hoc Dunn test with Bonferroni adjustments indicated that this difference was significant between 17 to 20 year olds and 21 to 24 year olds (*P* = 0.03). This suggests that the younger students in this sample were more comfortable with learning anatomical information online via pre‐recorded lectures than in‐person during anatomy laboratory practical sessions. All other items compared across age groups were insignificant (*P* > 0.05). There were no significant differences in scores between genders *χ* ^2^(2) = 0.168, *P* = 0.92, with a mean rank score of 45.21 for males, 47.68 for females, and 43.50 for other.

### Inductive content analysis of open‐ended reponses

Twenty‐six participants (27.95%) contributed to the open‐ended responses. Analysis of these responses revealed two categories: (1) benefits of pair learning, and (2) suggestions for improvement of the pair‐learning system (see Table 4). Five subcategories were also distinguished. For the category “benefits of pair‐learning,” students indicated that the pair‐based system provided them with greater hands‐on experience with the donor body and that the additional space in the anatomy laboratory enabled specific and valuable learning opportunities: “The pairing system gave us some good opportunities to learn more specific things and dissect what we wanted”, “So much hands‐on experience that we wouldn't get in bigger groups”. One student cited that “sacrificing the extra two hours” was beneficial in terms of ensuring “quality” preceded “quantity.”

**TABLE 4:** Inductive content analysis of open‐ended responses. Categories and subcategories are presented with supporting comments

Categories/subcategories	Supporting comments
**Benefits of pair‐learning**	
Greater one‐on‐one time with the donor body	*Working in pairs was really beneficial as we could look at the donor properly. But also, we could help each other with our learning* *I really feel that I benefitted from a pair‐based system. I had so much one‐on‐one time with the donor. Having talked to student in older years about their experiences with having 10–12 persons per donor, I am glad that a pair‐based system was used this year* *Pair based learning is good as you get to have a good look at the donor body* *While it was slightly inconvenient to have only one hour a week in the lab, I greatly enjoyed it and feel like I got a more hands‐on experience than my upperclassmen who were 8–10 per donor body did* *I cannot comment on what was going on before where it seems you were sharing a cadaver between 12 and had 3 h to dissect. I can only imagine that being in a pair for an hour gives you more time with the body* *Pairs are the best! So much hands‐on experience that we would not get in bigger groups. Definitely worth sacrificing the extra two hours. Quality over quantity* Pairing during anatomy practicals^a^ has allowed me to be more hands on with the cadaver and this really helps with learning
Extra space in the dissecting room	*The pair‐based system meant each person had to interact with the donor body consistently each week. If it was in a larger group I do not I would have had the same time or the same confidence for asking questions* *Even though there was just one hour of lab time per week, that fact that there were just two of us meant that there was more than enough time to see what we needed to see each week* *It was also very interesting to be able to walk around the dissection theatre to look at other donor bodies, which I feel would have been more difficult with more people in the room* *The pairing system gave us some good opportunities to learn more specific things and dissect what we wanted* *I much preferred working in pairs, as opposed to last year where we would have a lot more people crowding around the donor*
**Suggestions for improvement**	
Lack of opportunity to experience anatomical variation	*I would prefer to be able to move around and get a look at some anatomical variation between cadavers* *I thought the pair‐based learning was extremely useful, the only part that was not ideal was that we were only able to use one donor and so could not really see any variations*
More prosection less dissection	*We had to dissect some structures ourselves, which was very time‐consuming* *I wish more of the organs/tissues are dissected for better understanding* During this semester's TAP^b^ sessions, my cadaver was mostly not dissected so I could not see many structures
Additional Time	*I do believe we should have more time at the donor stations, like 2 h. If we were to maybe have 4 people to a donor and 2 h with a 5 min break in between I feel that would be the most optimal* *I wish we had more time in the anatomy lab to go over more content* *More hours in lab should be provided*

^a^ Practicals, refer to “practical sessions” in the anatomy laboratory.

^b^ TAP, refers to ‘Thorax, Abdomen and Pelvis’ anatomy.

A suggested area for improvement included more opportunities to experience anatomical variation “*we were only able to use one donor and so couldn't really see any variations*.” Due to social distancing guidelines imposed by the Covid‐19 pandemic, students were requested to stay at their designated donor stations to avoid unnecessary contact with other students, and movement around the anatomy laboratory was facilitated only under the supervision of an anatomy demonstrator. Students cited that they would “*prefer to be able to move around*” and observe “*some anatomical variation between cadavers*.” Others took initiative and requested this from their demonstrators: “*it was very interesting to be able to walk around the dissection theatre to look at other donor bodies, which I feel would have been more difficult with more people in the room”*. Another area for improvement included the need for cadavers to be dissected to a more detailed standard *“I wish more of the organs/tissues were dissected for better understanding*.”

## DISCUSSION

The aim of this study was to examine student perceptions of pair learning as a contemporary hybrid teaching method for thorax, abdomen, and pelvis cadaveric anatomy learning. This work demonstrated that first year medical students are satisfied with short one‐hour pair‐based anatomy practical sessions, supplemented with online pre‐ and post‐practical session learning resources. Although students recognized the merits of more time in the anatomy laboratory, including opportunities for self‐directed study and added exposure to anatomical variation, they felt that having two students per station enabled sufficient hands‐on time with the donor body and fostered learning opportunities that would not be possible with larger groups. Strong preferences for quality one‐on‐one time with the donor body supported by useful online resources suggest this modality should be a key consideration in course design for anatomy curricula.

### Dyad dynamics and factors that influence collaboration

This cohort of students generally expressed a positive view of their dyads' functionality during practical sessions. Students agreed that both they and their partners worked well together and that the pair‐based system helped their understanding of anatomy. Likewise, students indicated that both they and their partner were well‐prepared for the practical session each week. And although students were requested to stay at their respective stations to minimize social contact, students expressed that they still felt connected to their peers. Peer–peer interactions are known to be greatly influenced by personality and gender congruency. For instance, dyads with congruent levels of extroversion have been shown to interact more frequently (Wang et al., 2022), and “uncertainty reduction theory” proposes that similarity enhances friendship formation and maintenance therefore promoting cooperation while reducing stress and anxiety during peer–peer interactions (Basinger et al., 2020). While characteristics concerning personality type were not examined as part of this study, positive peer experiences may be associated with high frequencies of the conscientious personality type. The contentious personality type, which is included in the Big Five Personality Traits Model (Goldberg, 1990), has been shown to be a significant predictor of performance in medical school (Doherty & Nugent, 2011), and high conscientious individuals have been shown to produce higher grades in a gross anatomy course (Hintz et al., 2019). Further investigations into dyad dynamics and personality type as it relates to anatomy may be an interesting area of future inquiry.

Although approximately half of students in the study sample indicated that they would be interested in a surgical/radiological career, this did not appear to influence satisfaction with the dyad system. Such a finding is contrasted by Jeyakumar et al. (2020) who identified career interest as a positive predictor of regular attendance and participation in dissection, and thus positive experiences associated with practical sessions. The dyad system can therefore be considered an adaptive approach that encourages active participation in the anatomy laboratory and can accommodate for the strengths and weaknesses of each individual in a pair. Consequently, a supportive learning environment is achieved and discrepancies between student opinions of what is an effective use of time are lessened. Of relevance here may be grouping procedures, that is, how dyads were allocated. Methods for assigning student groups have been noted to affect the social structure of a classroom and thus learning. Notably, seminal educational research by O'Reilly and Illenberg (1969) expressed that student grouping based on hierarchical characteristics result in lower examination performance and more negative attitudes toward learning than diffuse classrooms (groupings of students with varying age profiles and heterogeneous academic capabilities). The higher the mean test score for any classroom group, the more diffuse the social structure (O'Reilly & Illenberg, 1969). In this present study, students were assigned to dyads alphabetically which makes it unlikely that a hierarchical structure based on academic performance was in effect. Nonetheless, based on high mean satisfaction scores within the study sample and that approximately 50% of students were surgical/radiological career focused, we can assume that many dyads were diffused pairings.

It must be acknowledged that although a hybrid dyad pedagogical approach with online resources enables students to meet the primary learning objectives, consideration must be given as to whether students are being underprivileged by other group dynamics that are so efficiently facilitated via small group learning (Bay et al., 2020). Members of dyads with differing experiences and strengths have repeatedly been shown to outperform similarly paired individuals in tasks of creativity and problem solving (Xue et al., 2018; Sun et al., 2021). Triads (groups of three persons) have also been shown to cover more content than dyads or students working independently (Spaulding, 1984). Thus, larger groups have more differing people, resulting in a greater pool of experiences and thus potentially better outcomes. A comparison across examination scores with students in dyads versus small groups may provide intriguing results and help to formulate more concrete understandings of the reciprocal and interactive capacity of dyad pedagogy.

At Trinity College Dublin, a great emphasis is placed on the human body donation program as a way of fostering healthy and professional relationships between students and their donor bodies. It was important that this teaching philosophy was maintained in an era of such widespread change. Weeks et al. (1995) has alluded to the relationship between student and cadaver as similar to that of the clinician and their patient and as such is one that fosters empathy and respect toward donors and future patients. Other writers have recognized that anatomy teaching is moving in a more humanistic direction with the growth of many medical schools offering commemoration services following the dissecting process (Ferguson et al., 2008; Pawlina et al., 2011; Jones et al., 2014; Jones & King, 2017). At our institution, cadavers are not anonymized, and students learn of the donor's name, partial medical history, and cause and date of death. Identification of donors can provide students with an opportunity to learn about and practice patient confidentiality. A concern with the dyad system among faculty was whether shorter practical sessions enabled this relationship to develop. As evidenced by participant responses, student expressed that a one‐hour practical sessions were sufficient in terms providing appropriate and adequate face‐to‐face time with their respective donors. Likewise, open‐ended responses reaffirmed that quality time rather than quantity time with the donor body enabled ample hands‐on time and this facilitated learning. In line with humanizing trends that are become increasingly evident in contemporary anatomy, the dyad approach is one that fosters rather than hinders this relationship.

### Transitioning from dissection to semi‐prosection and a view of student anatomical self‐efficacy

Cadaveric dissection is regarded by many anatomists as an unrivaled teaching method with benefits that extend far beyond the mere learning of anatomy (Winkelmann, 2007; Korf et al., 2008; Hu et al., 2018). It aligns well with modern medical education trends that promote collaborative work, ethical consciousness, and communication skills and must therefore be considered as an opportunity to nurture such graduate attributes (Rizzolo, 2002; Azer & Eizenberg, 2007; Sherman, 2008). Notwithstanding, limited curricular time, a lack of qualified anatomy demonstrators, and extrinsic factors such as that of the recent Covid‐19 pandemic are continually posing difficulties for this teaching modality. Anatomists' perceived benefits of dissection, however, are perhaps outweighed by the preferences of students. This current study found that some students maintain the view that dissection is an ineffective use of limited curricular time, and indeed previous student preference studies have shown that students generally believe prosection to be more efficient (Dinsmore et al., 1999; Davis et al., 2014; Dissabandara et al., 2015; Wisco et al., 2015). Similarly, data pertaining to examination performance has shown no superiority for dissection over prosection‐based curricula (Wilson et al., 2018; Williams et al., 2019; Lackey‐Cornelison et al., 2020). This communicates that student preference should continually be considered when designing teaching programs. Student preference must maintain its value in educational research and moreover be used appropriately and efficiently for informing educators on what is best suited to students' needs.

Students' lack of confidence in performing dissection is also evidenced by this study. Burgoon explains that this phenomenon can be referred to as “low anatomical self‐efficacy,” that is, an individual perceives within themselves an inability to successfully complete tasks such as dissecting, learning anatomical knowledge, and applying anatomical knowledge to clinical scenarios (Burgoon, 2008). Qualitative feedback from previous studies has reflected that reinforcing proper dissection techniques should be a priority during initial laboratory sessions to ensure that novice dissectors are equipped with the knowledge and skill to benefit from dissection, thus improving self‐efficacy (Jeyakumar et al., 2020). In this study, no such concerns were raised, it must therefore be inferred that such lack in confidence must instead be attributable to time constraints. Students indicated that they were dissatisfied with the semi‐prosected format because they *“couldn't see many structures”* and *“had to dissect some structures”* themselves which was *“very time consuming”* and prevented *“better understanding.”* It is possible that students who were not active in performing dissection did not acknowledge the immersive experience that dissection has to offer and, as a result, were somewhat less engaged and more likely to report negative experiences. Although the dyad approach presented here was executed at the cost of traditional prolonged dissecting time, semi‐prosection of cadavers was intended to provide students with the best of both worlds. That is, an opportunity to dissect with the advantage of having the majority of the dissection readily completed. Nonetheless, we uphold the view that active dissection should continue to be applied in practical sessions and take strength in the fact that it engages all three domains of learning; cognitive, affective, and psychomotor (Kuyatt & Baker, 2014; Hadie, 2018). However, the pressures imposed by social distancing and the additional anxieties that have coexisted for students learning anatomy during this pandemic need also be acknowledged. Students willing to dissect praised the semi‐prosected arrangement of cadavers, indicating that it gave them “*some good opportunities to learn more specific things and dissect what [they] wanted*”. Such students took authorship in their own learning, worked collaboratively, and reaped the benefits of an immersive experience allowing them and their partners to develop the competencies aforementioned, albeit in a highly time‐constrained environment. Arguably, these students may represent those interested in surgical or radiological disciplines as has been noted in previous student perception studies (McWatt et al., 2021). Although this 1‐h dyad approach is a reactive Covid‐19 measure to ensure students had the opportunity to dissect in a dissection‐based course, ‘semi‐prosection,’ as we have termed here, may be an interesting standpoint for future pedagogical research enabling students to reap the benefits of both dissection and prosection.

### Strain on academics and online resources

Applying dyad pedagogy in the laboratory meant that the approach was not reliant on the presence of additional demonstrators during practical sessions. Arguably, the approach can be seen to enable large groups of students to have very small‐group experiences with their educators which may not have otherwise been possible with additional students in the room. Students indicated that they were satisfied with the staff–student ratio and that large groups would have limited their confidence in asking questions. The strategy also enabled students to receive directive and facilitative feedback from demonstrators. “Directive feedback” was used to inform students of their knowledge shortfalls with the aim of enabling students to achieve their desired grades. “Facilitative feedback,” such as assisting students in developing their dissection technique or encouraging dyads to work collaboratively, was used to guide students on their professional developmental trajectories (Lachman, 2020). Demonstrators were positioned to guide and motivate their students both academically and professionally in a way that cannot be facilitated successfully with large student groups. The question remains however whether the dyad strategy puts extra strains on academics, demonstrators, and support staff. In terms of time spent teaching in the laboratory, the hours remain the same. Where one anatomy demonstrator was accessible to approximately 36 students over a three‐hour period prior to the pandemic, the same demonstrator was now accessible to eight students over a one hour period. An average of three hours was required by each demonstrator to prepare their teaching materials and complete semi‐prosections of their assigned cadavers. However, the weight of semi‐prosection was shared with technical staff and thus alleviated the additional strain placed on demonstrators. Notwithstanding, it must be acknowledged that all demonstrators regardless of previous experience, must revise for teaching sessions. Through the preparation of semi‐prosection, demonstrators were able to revise using cadavers, creating an opportunity to familiarize themselves with the variations and intricacies of each cadaver prior to student attendance in the laboratory. This represents a productive use of time, time that would otherwise have been spent reviewing atlases and models. This system may be particularly useful for medical demonstrators with surgical career intentions where time to study independently with a cadaver is greatly valued (Willan et al., 1992). Likewise, the process of prosection may be beneficial for near‐peer tutors as way of providing deeper learning of anatomy through teaching (Evans & Cuffe, 2009).

The use of online resources represents a multimodal approach to learning anatomy. Although the Covid‐19 pandemic has accelerated the use of a blended approaches to learning (Bao, 2020; Mukhtar et al., 2020), in anatomy, this is not a new conception. In their critical review on best teaching practices in anatomy education, Estai and Bunt (2016) state that “no single teaching tool has been found to meet curriculum requirements” and propose that the best way to teach modern anatomy is by combining multiple pedagogical resources to complement one another. Multimodal approaches have received support from other anatomists, particularly for those that supplement in‐person sessions with online quizzes and activities (Rizzolo et al., 2010). In this current study, there was prodigious agreement from students that the pre‐ and post‐practical session activities were useful tools for private study and revision indicating that a transition to hybrid learning may be beneficial for anatomy. Using a strength, weakness, opportunity, threat (SWOT) analysis, Longhurst et al. (2020) identified “incorporation of blended learning in future curriculum development” as the most frequently cited opportunity by anatomy faculty in the United Kingdom and Republic of Ireland in response to the Covid‐19 pandemic. Academics also suggested that the pandemic presented them with an opportunity to develop resources for upcoming years, allowing them to integrate blended learning techniques into their curricula. Other reviews of blended learning approaches in anatomy have reported that such techniques improve not only academic performance but also motivation, attitude, and enhance learning experiences (Liew et al., 2015; Khalil et al., 2018). These studies, together with our finding that students perceive online resources as valuable, creates a need for anatomists and academics to prioritize time to create high‐quality blended learning resources.

### Limitation of the study

Limitations of the current study include its cross‐sectional nature and a potential straight‐lining response bias that can often be associated with agree/disagree questionnaire matrices. The study only assessed student perception which is a subjective measure and can be swayed by experience. Despite this, anonymity was maintained which increases the validity of the findings. Anonymity, however, prevented comparisons between perceptions and examination performance which may have been beneficial in terms of comparing perceptions of group dynamics across student cohorts. Although questions were modeled on previously published studies, the questionnaire did not comprehensively examine all aspects of dyad pedagogy. Such aspects could have been identified by utilizing student focus groups and pilot testing. Factors identified in the questionnaire revealed low Cronbach alpha values. Each of the factors could have been strengthened through revision and rewriting of items with lower primary loadings. Future studies investigating longitudinal changes in student perception toward dyad pedagogy as they transition into their second preclinical year and thereafter may provide key insights into knowledge retention and the importance of teamwork and collaboration. The cost and time associated with preparing semi‐prosections may be a limitation to the implementation of short one‐hour pair‐based practical sessions across medical schools. Finally, the conclusions of this study reflect a first‐year preclinical medical program during an unprecedented worldwide pandemic with high student anxiety and thus must be evaluated in this context.

## CONCLUSIONS

In summary, this article examines student perceptions of short one‐hour pair‐based anatomy practical sessions supplemented with hybrid online learning resources as it relates to thorax, abdomen, and pelvis anatomy. Data from this study indicate that students rate online pre‐ and post‐practical session learning resources as valuable and are generally satisfied with pair learning as a pedagogical method in the anatomy laboratory. The observed trend in anatomical education is that there is a consistent increase in transactional distance between students and their educators. This issue may be ameliorated by pair‐learning strategies which allows a large group of students to have small group experiences with their educators. Together with the ongoing climate of diminishing devoted time to anatomy, these results highlight the indispensability of student perception and the importance of evidence‐based pedagogy.

## Supporting information


supinfo
