# Supplementary material for: Dyad pedagogy in practical anatomy: A description of the implementation and student perceptions of an adaptive approach to cadaveric teaching
Source: Anat Sci Educ. 2022 May 27;16(1):99–115. doi: 10.1002/ase.2184 (PMC10084083; doi:10.1002/ase.2184)
Supplement: Supplementary file 1 — supinfo [file ASE-16-99-s001.docx]

**APPENDIX**

**Questionnaire**

Q1: Are you:

- Male
- Female
- Other

Q2: Please specify your age profile:

- 17 - 20
- 21 - 24
- 25 - 28
- 29 +

Q3: Prior to starting your studies in Trinity College Dublin, did you have a third level anatomy education?

- Yes
- No

Q4: Prior to starting your studies in Trinity College Dublin, did you have experience with cadaveric anatomy?

- Yes
- No

Q5: What is your current career interest?

- Surgical/Radiological
- Other

Q6: How many Thorax, Abdomen, and Pelvis (TAP) practical sessions did you attend?

- 0-4
- 5-8

Directions: Please indicate to what extent you agree or disagree with the following statements: A = Strongly agree, B = Agree, C = Neither agree nor disagree, D = Disagree, and E = Strongly disagree

| 1. | I was well prepared for my practical each week | A | B | C | D | E |
| --- | --- | --- | --- | --- | --- | --- |
| 2. | Given the paired nature of the anatomy practicals, I still feel connected to my other classmates | A | B | C | D | E |
| 3. | During the pair-based anatomy practicals, I had enough face-to-face time with my demonstrator | A | B | C | D | E |
| 4. | During the pair-based anatomy practicals, I had enough hands-on time with the donor body (cadaver) at my station | A | B | C | D | E |
| 5. | I learn better from the online lectures than in my pair-based anatomy practicals | A | B | C | D | E |
| 6. | The pre- and post-lab learning activities on Blackboard were useful tools for private study and revision | A | B | C | D | E |
| 7. | Given the one-hour pair-based anatomy practicals, I still feel the practical examination process provides a fair assessment | A | B | C | D | E |
| 8. | My anatomy lab partner was well prepared for the practical each week | A | B | C | D | E |
| 9. | My anatomy lab partner and I worked well together | A | B | C | D | E |
| 10. | Pair-based learning helped my understanding of anatomy | A | B | C | D | E |

Directions: Please indicate your answer: A = Yes, B = No, and C = Not sure

| 11. | During the Thorax, Abdomen, and Pelvis practicals, I saw the Left Anterior Descending Artery (aka: the anterior interventricular branch of the left coronary artery) on the donor body at my station | A | B | C |
| --- | --- | --- | --- | --- |
| 12. | During the Thorax, Abdomen, and Pelvis practicals, I saw the major duodenal papilla on the donor body at my station | A | B | C |

Directions: Please indicate your answer: A = Yes, B = No, C = Prefer not to say, and D = Not applicable, I did not have a lab partner

| 13. | Did you enjoy working in pairs for your Thorax, Abdomen, and Pelvis practicals? | A | B | C | D |
| --- | --- | --- | --- | --- | --- |

Directions: Please leave any other thoughts

|  |
| --- |
